# Circulating microRNA as Biomarkers for Gestational Diabetes Mellitus—A Systematic Review and Meta-Analysis

**DOI:** 10.3390/ijms24076186

**Published:** 2023-03-24

**Authors:** Sofie Dinesen, Alisar El-Faitarouni, Nanna Lond Skov Frisk, Anja Elaine Sørensen, Louise Torp Dalgaard

**Affiliations:** 1 Department of Science and Environment, Roskilde University, 4000 Roskilde, Denmark; 2 Roskilde Hospital, Region Zealand, 4000 Roskilde, Denmark

**Keywords:** gestational diabetes, microRNA, diagnostic biomarker, prognostic biomarker

## Abstract

Gestational diabetes mellitus (GDM) is a severe pregnancy complication for both the woman and the child. Women who suffer from GDM have a greater risk of developing Type 2 diabetes mellitus (T2DM) later in life. Identification of any potential biomarkers for the early prediction of gestational diabetes can help prevent the disease in women with a high risk. Studies show microRNA (miRNA) as a potential biomarker for the early discovery of GDM, but there is a lack of clarity as to which miRNAs are consistently altered in GDM. This study aimed to perform a systematic review and meta-analysis to investigate miRNAs associated with GDM by comparing GDM cases with normoglycemic controls. The systematic review was performed according to PRISMA guidelines with searches in PubMed, Web of Science, and ScienceDirect. The primary search resulted in a total of 849 articles, which were screened according to the prior established inclusion and exclusion criteria. Following the screening of articles, the review was based on the inclusion of 35 full-text articles, which were evaluated for risk of bias and estimates of quality, after which data were extracted and relative values for miRNAs were calculated. A meta-analysis was performed for the miRNA species investigated in three or more studies: MiR-29a, miR-330, miR-134, miR-132, miR-16, miR-223, miR-155, miR-122, miR-17, miR-103, miR-125, miR-210, and miR-222. While some miRNAs showed considerable between-study variability, miR-29a, miR-330, miR-134, miR-16, miR-223, and miR-17 showed significant overall upregulation in GDM, while circulating levels of miR-132 and miR-155 were decreased among GDM patients, suggesting further studies of these as biomarkers for early GDM discovery.

## 1. Introduction

Gestational diabetes mellitus (GDM) is diabetes occurring during pregnancy and causes serious complications for both the woman and the baby. Women who develop GDM are at high risk of developing Type 2 diabetes mellitus (T2DM) later in life or recurrent GDM in future pregnancies. GDM has a significant risk of complications for both mother and child [1]. Both during pregnancy and immediately following delivery, there is an increase in morbidity: preeclampsia and maternal mortality are at an elevated risk, and GDM fetuses are also more likely to experience macrosomia, early birth, neonatal icterus, and perinatal hypoglycemia [2]. Early intervention may prevent the development of perinatal morbidity. Several risk factors that may contribute to the development of GDM in women have been identified. These include maternal age, Anglo-European ethnicity, smoking, family history of diabetes, and previous birth of a child with macrosomia, as well as obesity [2]. Considering this, the International Diabetes Federation (IDF) and the International Associations for the Diabetes in Pregnancy Study Groups (IADPSG) advise screening for GDM, preferably by measuring plasma glucose levels (HbA1c, random or fasting plasma glucose values) in all or high-risk women at the first antenatal visit, followed by screening using an oral glucose tolerance test (OGTT) at gestational week 24–28.

Thus, efforts are underway to identify better biomarkers that will enable the diagnosis of GDM at an earlier stage or provide more precise prognostic predictions of the risk of GDM. Circulating RNAs, especially microRNAs (miRNAs), have been suggested as novel biomarkers for the early detection of GDM. MiRNAs are small noncoding single-stranded RNA molecules of about 22 nucleotides in length. Found in plants, animals, viruses, human tissues, and blood, miRNAs function by RNA silencing and post-transcriptional regulation of gene expression. Their primary role is, therefore, to regulate messenger RNA (mRNA) degradation and to adjust protein levels. More than 2500 miRNAs have been identified in the human genome, and miRNAs regulate at least 30% of protein-coding genes [3,4,5]. In addition to being located intracellularly and controlling gene expression, miRNAs are also secreted to the extracellular environment. Increasing evidence suggests that cells can package miRNA and other RNA species in exosomes or microvesicles before releasing them into the bloodstream [6]. Circulating RNAs are promising candidates for use as biomarkers because of their widespread distribution in bodily fluids, such as serum, plasma, urine, sweat, and saliva [6,7]; their protection by AGO2 and other proteins [8,9,10], or by lipid bilayer membranes in extracellular vesicles (EVs); and the fact that levels of particular miRNAs have been found to reflect various pathophysiological conditions.

Concerning GDM, multiple studies have investigated the circulating levels of a considerable number of specific miRNA species in relation to GDM, sometimes with incongruent results. Biomarker studies of circulating RNAs often study a low number of subjects, which carries the risk of bias in reporting and generally low power [11]. Thus, the current study aimed to perform a systematic review and meta-analysis of circulating miRNA with respect to early detection of GDM with the overall purpose of identifying a credible set of consistent GDM-associated miRNAs. Specifically, the aim was to identify a credible set of circulating miRNAs confidently associated with GDM.

## 2. Materials and Methods

We followed PRISMA guidelines for the systematic review [12,13]. Details of the protocol for this systematic review were registered on PROSPERO and can be accessed at www.crd.york.ac.uk/PROSPERO/display_record.asp?ID=CRD42021241518 (accessed on 9 April 2021) [14]. The PRISMA checklist and PRISMA abstract checklists are enclosed as Supplementary Tables S1 and S2.

### 2.1. Data Extraction

**Figure 1:**
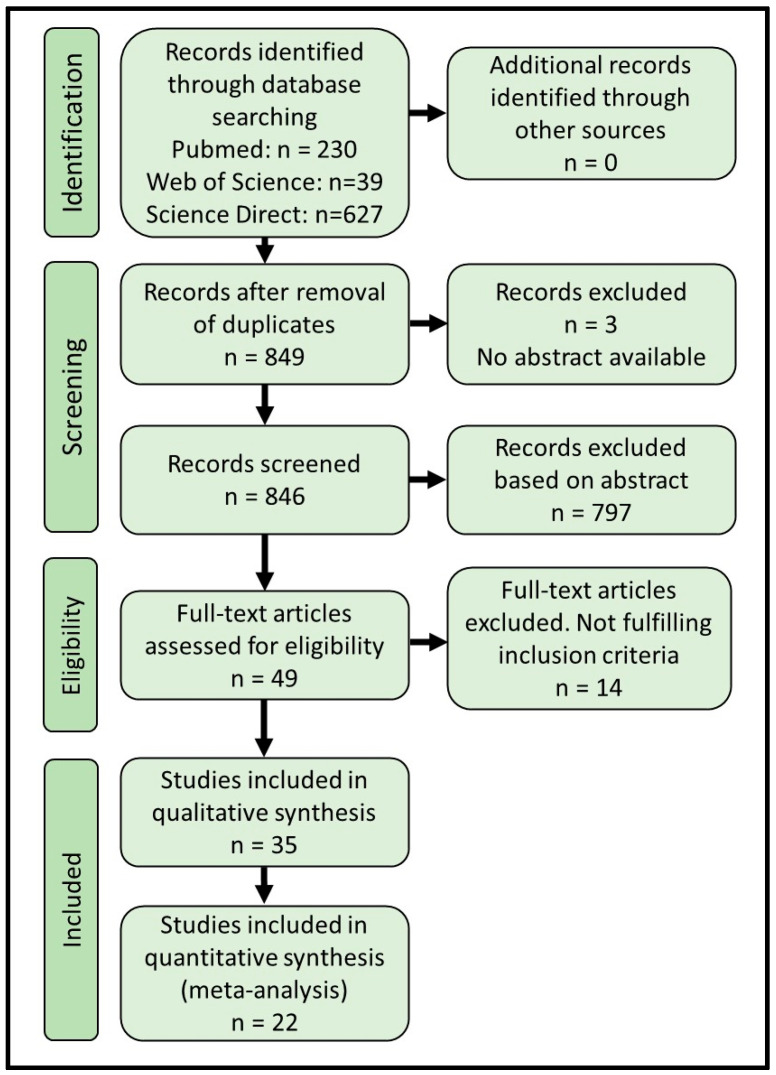
PRISMA flow diagram showing the search and inclusion numbers of the systematic review.

The literature search was performed using the search string: (Gestational diabetes OR pregnancy-induced diabetes) AND (microRNA OR miRNA OR microribonucl*) AND human, in the databases PubMed (https://pubmed.ncbi.nlm.nih.gov/ (accessed on 22 November 2022), National Library of Medicine, National Institute of Health, Bethesda, MD, USA), ScienceDirect (Elsevier, Amsterdam, The Netherlands), and Web of Science (Clarivate, London, UK). Searches were updated in November 2022. All abstracts were independently screened manually by two people to evaluate inclusion, and any disagreement was resolved by discussion with a third evaluator. We included observational studies, case-control studies, cross-sectional studies, and cohort studies. Only studies including at least one GDM group and a nondiabetic control group were included. Studies were included regardless of body weight or adiposity, gestational age, or ethnicity. Only studies measuring circulating miRNA through quantitative methods, such as reverse-transcription quantitative PCR, RNA-sequencing or microarrays were included. We excluded studies of twin pregnancies, or study groups in which subjects had a prior diagnosis of either Type 1 or Type 2 diabetes mellitus. Moreover, only peer-reviewed original articles using English language were included (Figure 1).

We performed extensive data extraction on the included articles to extract publication information, identity of miRNA measured, number of subjects in each group, information about adiposity of investigated subjects, GDM diagnostic criteria, gestational age at diagnosis, gestational age at sampling, sample material, measuring method for miRNA, reference gene used, measurement units, and measured levels and variation of examined miRNAs in studied cohorts (Supplementary Materials File S1). The software WebPlot digitizer, version 4.5 2021; https://automeris.io/WebPlotDigitizer/ (accessed on 22 November 2022) was used to extract levels of miRNA and degree of variation (SD, SEM, or 25 and 75 percentiles). Reported levels and degree of variation (SD) for each miRNA in the GDM group were recalculated relative to the control group, which was set to one. In some studies, the median instead of mean was reported. To extract data for the meta-analysis, we assumed normally distributed data for the calculation of SD, although some miRNAs were not normally distributed. Formulas used to calculate SD based on SEM or box plots are given in Supplementary Materials File S1.

### 2.2. Estimation of Study Quality

**Figure 2:**
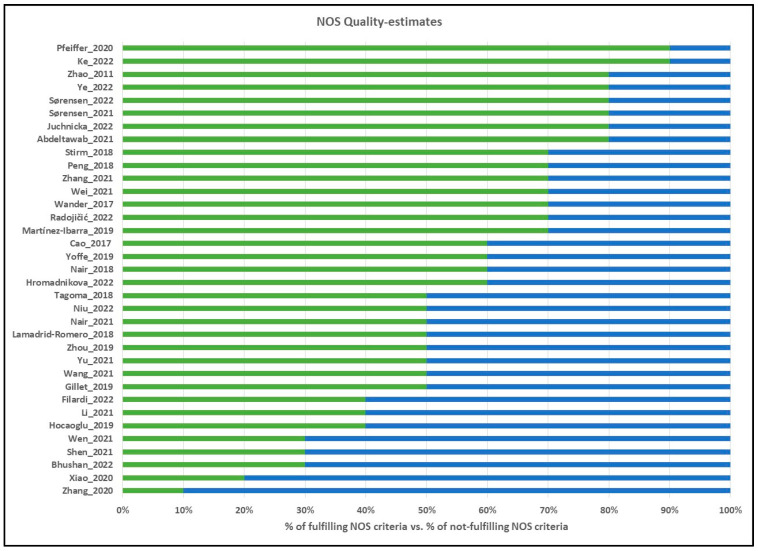
Quality evaluations of the included studies according to the NOS Scale. Green scale (right side) corresponds to the percentage of NOS scale points fulfilled, and blue scale (left side) corresponds to the percentage of NOS scale points not fulfilled. References for evaluated studies: Pfeiffer et al. (2020) [16], Ke et al. (2022) [17], Zhao et al. (2011) [18], Ye et al. (2022) [19], Sørensen et al. (2022) [20], Sørensen et al. (2021) [21], Juchnicka et al. (2022) [22], Abdeltawab et al. (2022) [23], Stirm et al. (2018) [24], Peng et al. (2018) [25], Zhang et al. (2021) [26], Wei et al. (2021) [27], Wander et al. (2017) [28], Radojičić et al. (2022) [29], Martinez-Ibarra et al. (2019) [9], Cao et al. (2017) [30], Yoffe et al. (2019) [31], Nair et al. (2018) [32], Hromadnikova et al. (2022) [33], Tagoma et al. (2018) [34], Niu et al. (2022) [35], Nair et al. (2021) [36], Lamadrid-Romero et al. (2018) [37], Zhou et al. (2019) [38], Yu et al. (2021) [39], Wang et al. (2021) [40], Gillet et al. (2019) [41], Li et al. (2021) [42], Hocaoglu et al. (2019) [43], Wen et al. (2021) [44], Shen et al. (2021) [45], Bhushan et al. (2022) [46], Xiao et al. (2020) [47], Zhang et al. (2020) [48].

Quality estimates were based on the Newcastle–Ottawa Scale [15], with the questionnaire adjusted for gestational diabetes (Figure 2, Supplementary Materials File S1).

### 2.3. Data Analysis and Statistics

All data are included in Supplementary Materials File S1. Qualitative analyses were made by sorting studies by sample materials tested, ethnicity, measurement methods, and time of measurement during gestation. We qualitatively assessed if these variables could be underlying sources of study heterogeneity. Meta-analysis for the relationship between miRNA levels and GDM diagnosis was performed on miRNAs reported with quantitative data in at least 3 studies. Several studies made comparisons at several time points during gestation for the same subjects. In order to not include the same subjects more than once per meta-analysis, we included the earliest measured time point only. Meta-analysis was performed using the R-package ‘*meta*’ [49] with the fixed-effects model, as most studied miRNA species were investigated in only a few studies. Between-study heterogeneity, τ^2^, was computed as the restricted maximum-likelihood estimator [50] as default in ‘*meta*’. Biases in meta-analyses were estimated by inspecting funnel plotsand outliers were inspected manually. For statistical evaluation, we used R and R-studio software (Vs. 4.2.2 and 2022.07.2, respectively) and the package ‘*meta*’. Excel 365 was used to organize data. *p*-values < 0.05 were considered significant.

## 3. Results

### 3.1. Data Inclusion

The systematic review followed PRISMA guidelines [12,13]. PRISMA abstract and article checklists for the current study are completed and available in Supplementary Tables S1 and S2. Through systematic searches in PubMed, Science Direct, and Web of Science, we identified 896 abstracts, with 849 remaining after removal of duplicates (Figure 1). We screened 846 records (3 were unavailable) and excluded 797 based on abstract content, leaving 49 articles for full-text screening, where 14 articles were excluded during full-text examination. In total, we were able to include 22 articles in the meta-analysis (Table 1) and an additional 13 articles in the qualitative part of the systematic review (Table 2), giving a final number of 35 original articles on which the systematic review and meta-analysis were based (Figure 1).

### 3.2. Quality Evaluation of Included Studies

The 35 included studies (Table 1 and Table 2) were evaluated using the Newcastle–Ottawa Scale (NOS) [15,54], with evaluation criteria listed in Supplementary Materials File S1, and the NOS evaluations for each included study are displayed in Figure 2. The average total NOS Score was 59%, ranging from 20% [47] to 90% [17]. The diagnosis of GDM was made using national guidelines or IADPSG criteria in 88% of studies (Criteria 3A), while none of the studies clearly described any loss of samples during experiments or data analysis (i.e., failed reactions, eliminated outliers, or absent values; Criteria 3C), although some studies did report number of subjects analyzed for each miRNA.

### 3.3. Characterization of Patient Populations among Studies

Patient characteristics were extracted from all included studies to investigate overall heterogeneity regarding included patients. Ten studies diagnosed GDM using IADPSG criteria [9,19,20,21,24,29,31,33,43,55], seven used ADA criteria [17,18,25,28,34,37,38], three used national guidelines [16,36,51], and one study used WHO criteria [22], while the remaining studies did not define GDM diagnosis according to any explicit diagnostic criteria (Supplementary Materials; Data Extraction Sheet). Patients were generally included with no specific reference to the BMI of patients and control subjects; however, four studies only investigated obese or overweight subjects [18,20,21,37], and one study only investigated subjects with a BMI < 25 kg/m^2^ [23]. In 21 (60%) of the studies, the control group was explicitly matched to the GDM group with respect to BMI. The majority of studies examined subjects in the second trimester (weeks 24–28), but four studies examined subjects in the first trimester [9,21,41,47]. Thus, the included studies were comparable overall, although diagnostic criteria were only explicit in 50% of the studies [16].

### 3.4. Qualitative Analysis of Experimental Approaches for miRNA Quantification

The sample material varied between plasma (32%) [25,28,29,30,31,33,34,35,51], serum (53%) [9,16,17,18,20,21,22,23,37,38,43,46,47,48,55], whole blood [24], and EVs (14%) [19,36,41,52]. Circulating miRNAs were quantified using a variety of approaches, such as discovery studies using small RNA sequencing followed by single miRNA reverse transcription–quantitative PCR (RT-qPCR) validation of selected miRNAs, RT-qPCR arrays, or candidate panels of single RT-qPCR assay, where RT-qPCR was by far the most used methodology.

RT-qPCR data are routinely analyzed using one to several reference RNAs, against which the target RNAs are compared. The most common references used are the U6 small nucleolar RNA (used in 13 out of the included studies), which are endogenously present, and *C. elegans* (cel) miR-39-3p (cel-miR-39, used in 10 out of the included studies), which is added as a spike-in during RNA extraction or cDNA synthesis. Additional endogenously present references used for data normalization were miR-423-3p, miR-454, beta-actin, miR-103a-3p, U58A and U36B small nucleolar RNAs, and miR-16. Other synthetic RNAs used as spike-in materials included *A. thaliana* miR-159, cel-miR-54, and cel-miR-238. One study did not report the normalization strategy for RT-qPCR data [31]. Only 4 original studies out of 30 included studies employed normalization to the average of several references, although this is recommended by guidelines for circulating RNA assays in clinical sciences [56]. Of note, levels of circulating miR-16 and miR-103 were previously reported to be associated with GDM [20,21,30,57], indicating that studies incorporating these two miRNAs as endogenous references [22,46] could generate biased data and complicate interpretations in the field. Thus, significant heterogeneity was identified regarding the use of references during data normalization for RT-qPCR studies, although spike-in RNAs derived from plant or nematode miRNA sequences as well as the endogenously expressed U6 RNA were used in many of the studies.

### 3.5. Diversity in Investigated Circulating miRNAs in Relation to GDM

**Figure 3:**
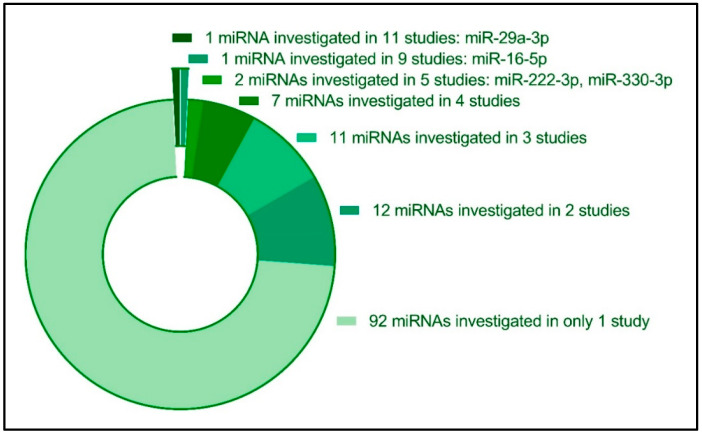
Diversity in the reported circulating miRNAs investigated in relation to GDM pregnancies. Twenty-two miRNAs were reported to be investigated in three or more original studies, while the majority of investigated miRNAs (*n* = 92 miRNA species) were only investigated in one original study.

We evaluated the number of times each miRNA was studied in relation to GDM (Figure 3). The majority of circulating miRNAs (*n* = 92) were only investigated in one study, suggesting that more studies of these particular miRNAs could potentially verify these miRNAs as being associated with a GDM diagnosis. Twelve miRNAs were each investigated in two studies (miR-126-3p [28,34], miR-137-3p [25,37], miR-142-3p [22,34], miR-142-5p [24,34], miR-191-5p [34,37], miR-195-5p [33,34], miR-21-3p [28,43], miR-224-5p [16,52], miR-27b-3p [28,52], miR-30c-5p [34,37], miR-517-5p [28,41], and miR-9-5p [9,37]. Eleven different miRNAs were each investigated in three original studies (miR-103-3p [16,20,21], miR-125b-5p [18,34,37], miR-134-5p [17,20,21], miR-19a-3p and miR-19b-3p [24,30,34], miR-20a-5p [30,33,34], miR-221-3p [33,34,35], miR-30-5p [34,37,41], miR-342-3p [33,34,41], miR-423-5p [19,34,52] and miR-92a-3p [33,34,52], while seven miRNAs were each investigated in four studies: Five of these were able to enter the quantitative meta-analysis and are covered below (miR-122-5p, miR-132-3p, miR-155-5p, miR-17-5p, miR-210-3p), while additional two miRNAs were investigated in four studies each, but for which not sufficient studies could enter the meta-analysis (miR-23a-3p [31,33,34,46], miR-99a-5p [18,19,34,52]) (Figure 3, Supplementary Materials File S1).

### 3.6. miR-29a

**Figure 4:**
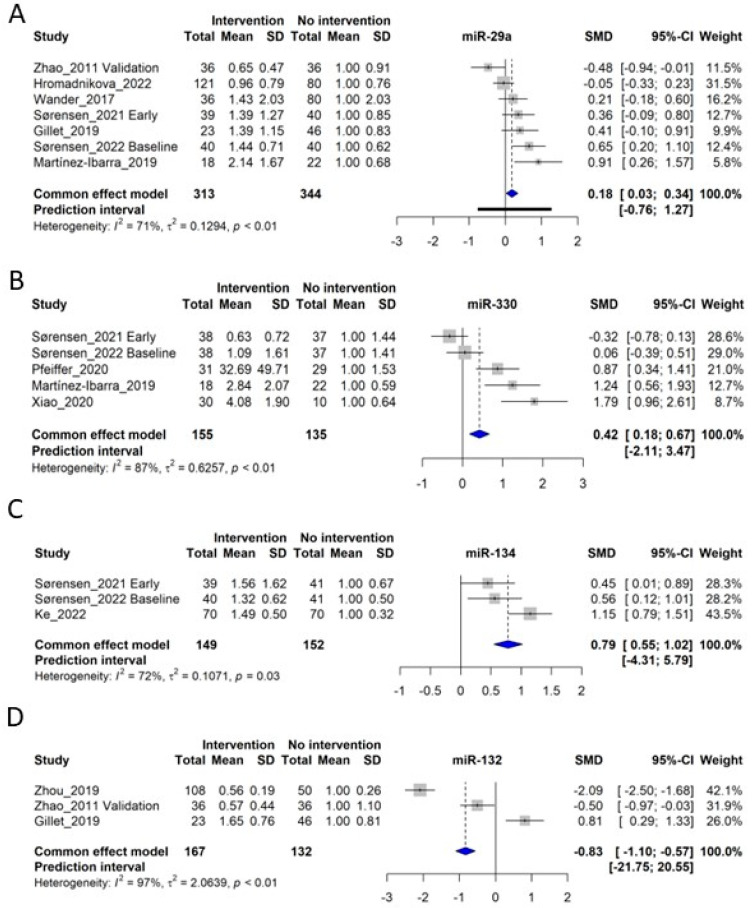
Forest plots of the fixed-effect meta-analysis of (**A**) miR-29a [9,18,20,21,28,33,41], (**B**) miR-330 [9,16,20,21,47], (**C**) miR-134 [17,20,21], and (**D**) miR-132 [18,38,41].

Seven original studies investigated circulating miR-29a [9,18,20,21,28,33,41] for which data were available for inclusion in the meta-analysis (*n* = 313 control subjects and *n* = 344 GDM patients) (Figure 4A). Overall, miR-29a was significantly increased in GDM patients, with a standardized mean difference (SMD) of 0.18, corresponding to a fold-increase of 1.18 in GDM patients (confidence interval (CI): 0.03–0.34, *p* < 0.05). However, significant heterogeneity was observed (*I*^2^ of 71%, τ^2^ = 0.1294, and *p* < 0.01), giving a prediction interval ranging from −0.76 to 1.27 (Figure 4A). Furthermore, Tagoma et al. (2018) [34] observed a three-fold increased miR-29a in GDM patients, further underlining that miR-29a is generally increased in circulation in GDM. Interestingly, Wander et al. (2017) [28] observed that circulating levels of miR-29a were associated with an increased risk of GDM, primarily if the fetus was male. Moreover, the closely related miR-29b was also observed to have increased in circulation [34,41]. It should be noted that the study by Zhao et al. (2011) [18], displaying a decrease in circulating miR-29a levels, is the only study investigating miR-29 in Chinese women, whereas the other studies were performed in European or Hispanic subjects.

### 3.7. miR-330

miR-330 was investigated in 5 original studies [9,16,20,21,47], for which the meta-analysis included 155 control subjects and 135 GDM patients. The meta-analysis showed an SMD of 0.42, corresponding to a fold-increase of 1.42 in GDM patients (CI: 0.18–0.67, *p* < 0.05), although significant heterogeneity was observed (*I*^2^ of 87%, τ^2^ = 0.6257, and *p* < 0.01), giving a prediction interval ranging from −2.11 to 3.47 (Figure 4B). There is a tendency that in early GDM pregnancies, miR-330 levels are slightly downregulated [20,21], and in late GDM, levels are upregulated [9,16,47]. Moreover, miR-330 was also determined to cause INS-1 clonal beta-cell dysfunction, suggesting possible functional effects of circulating miR-330 levels [47].

### 3.8. miR-134

Three independent studies investigated circulating miR-134 in connection to GDM [17,20,21], giving a total of 149 control subjects and 152 GDM patients for the analysis. The meta-analysis yielded an SMD of 0.79, corresponding to a fold increase of 1.79 in GDM (CI: 0.55–1.02, *p* < 0.05), with significant heterogeneity (*I*^2^ of 72%, τ^2^ = 0.1071, and *p* = 0.03), rendering a prediction interval of −4.31–5.79 (Figure 4C). Interestingly, miR-134 is located in a miRNA cluster in an imprinted region on Chr14q32, which is enriched in beta-cells and controls beta-cell proliferation [58].

### 3.9. miR-132

miR-132 was investigated in 4 studies [18,34,38,41], of which 3 entered meta-analysis [18,38,41], including 167 control subjects and 132 GDM patients. Unlike miR-29a, miR-330 and miR-134, miR-132 was found to be lower overall in GDM patients with an SMD of −0.83 (CI: −1.10 to −0.57, *p* < 0.05), also with considerable heterogeneity (*I*^2^ of 97%, τ^2^ = 2.0639, and *p* < 0.01). Gillet et al. (2019) measured miR-132 in extracellular vesicles, which might explain the difference from the two other studies (Figure 4D) [41].

### 3.10. miR-16

**Figure 5:**
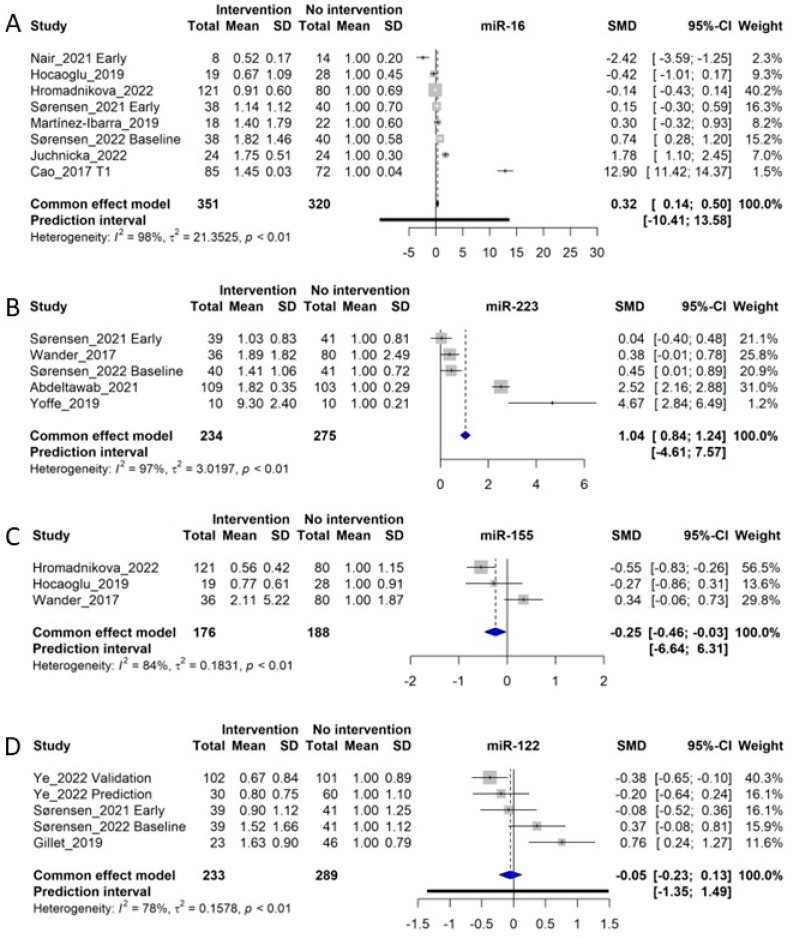
Forest plots of the fixed-effect meta-analysis of (**A**) miR-16 [9,20,21,22,30,33,36,43], (**B**) miR-223 [20,21,23,28,31], (**C**) miR-155 [28,33,43], and (**D**) miR-122 [19,20,21,41].

Eight studies of circulating miR-16 were included in the meta-analysis [9,20,21,22,30,33,36,43]. Qualitatively assessed, the analysis by Cao et al. (2017) [30] constitutes an outlier, as miR-16 was found to have a ~15-fold increased in GDM women, corresponding to an SMD of 12.9, while the remaining studies displayed SMDs from −2.42 to 1.78 (Figure 5A). However, as we detected no objective methodological differences between Cao et al. (2017) and the remaining studies, we did not exclude it from the analysis. A total of 351 GDM women and 320 control women entered the meta-analysis, for which the overall SMD was 0.32 (CI: 0.14–0.50, *p* < 0.05), similarly to other miRNAs with large heterogeneity (*I*^2^ of 98%, τ^2^ = 21.3625, and *p* < 0.01). Sørensen et al. (2022) demonstrated a significant upregulation of miR-16 in early diagnosed GDM women compared with normal glucose-tolerant (NGT) women, however, with an increase in miR-16 in weeks 35–37 increasing the difference with the control group [20]. miR-16, together with miR-29a and miR-134, all measured early in pregnancy, could predict GDM diagnosed in week 24–28, with an ROC AUC of 0.717 [21]. However, based on meta-analyses for miR-29a and miR-134, these two miRNAs appear to be more consistently associated with GDM compared with miR-16 (Figure 4A,D and Figure 5A).

### 3.11. miR-223

We identified 5 studies for inclusion in the meta-analysis of circulating miR-223, including 234 control subjects and 275 GDM patients [20,21,23,28,31]. The meta-analysis of the studies showed an SMD of 1.04, corresponding to a fold-increase of 2.04 in GDM patients (CI: 0.84 to 1.24, *p* < 0.05), although with a significant heterogeneity (*I*^2^ of 97%, τ^2^ = 3.0197, and *p* < 0.01), giving a prediction interval of −4.61–7.57 (Figure 5B). The observed heterogeneity is likely influenced by the study of Yoffe et al. (2019) [31], which demonstrated a much larger SMD of 4.67 compared with the mean of the other studies (average SMD of 0.85), although the weight of this study is low (1.2%). Overall, the meta-analysis data indicate that miR-223 is significantly upregulated during GDM, which correlates well with the fact that miR-223-3p has been associated with impaired insulin sensitivity in adipose tissues [59] and an upregulation in beta-cells during diabetes [60]. Moreover, circulating miR-223 seem to be increased in GDM women regardless of the time point of measurement during gestation, suggesting that miR-223 might also be increased in circulation prior to conception in these women. Of note, circulating EV levels of miR-223 were found to predict development of Type 2 diabetes from impaired glucose tolerance [61].

### 3.12. miR-155

In total, 3 original studies examined miR-155 [28,33,43] to include a total of 176 controls and 188 GDM subjects in the meta-analysis, yielding a combined SMD of −0.25 (CI: −0.46 to −0.03) with substantial heterogeneity (*I*^2^ of 84%, τ^2^ = 0.1831, and *p* < 0.01). Overall, this resulted in the prediction interval ranging from −6.64 to 6.31 (Figure 5C). While the overall result of the meta-analysis shows that low levels of miR-155 are associated with GDM, the study by Tagoma et al. (2018) [34] also found that levels of miR-155 were increased in GDM patients, adding to the assessment of heterogeneity for this miRNA. Thus, miR-155 is not suitable as a biomarker for GDM. As late pregnancy was measured in all three included studies, its function as a diagnostic biomarker could not be considered.

### 3.13. miR-122

Circulating miR-122 was examined in 4 different studies (5 cohorts) in relation to GDM, giving a total of 233 control and 289 GDM women [19,20,21,41]. The meta-analysis showed an SMD of −0.05 (CI: −0.23 to 0.13), with an observation of substantial heterogeneity (*I*^2^ of 78%, τ^2^ = 0.1578, and *p* < 0.01), resulting in a prediction interval of −1.35–1.49 (Figure 5D). All the studies investigated miR-122 in early pregnancy, corresponding to a gestational week before week 20. However, no clear tendency can be seen across the studies, and therefore miR-122 is estimated to have little to no correlation with the development of GDM. Circulating miR-122, being released from hepatocytes during impaired glucose tolerance and in states of fatty liver disease [62], is an independent predictor of progression from impaired glucose tolerance to Type 2 diabetes [63]. However, data for patients with GDM regarding miR-122 display considerable heterogeneity, which cannot be explained by ethnicity, degree of obesity, biofluid material or different use of reference genes (Supplementary Materials).

### 3.14. Additional miRNAs Undergoing Meta-Analysis: miR-17, miR-103, miR-125a/b, miR-210, and miR-222

#### 3.14.1. miR-17

**Figure 6:**
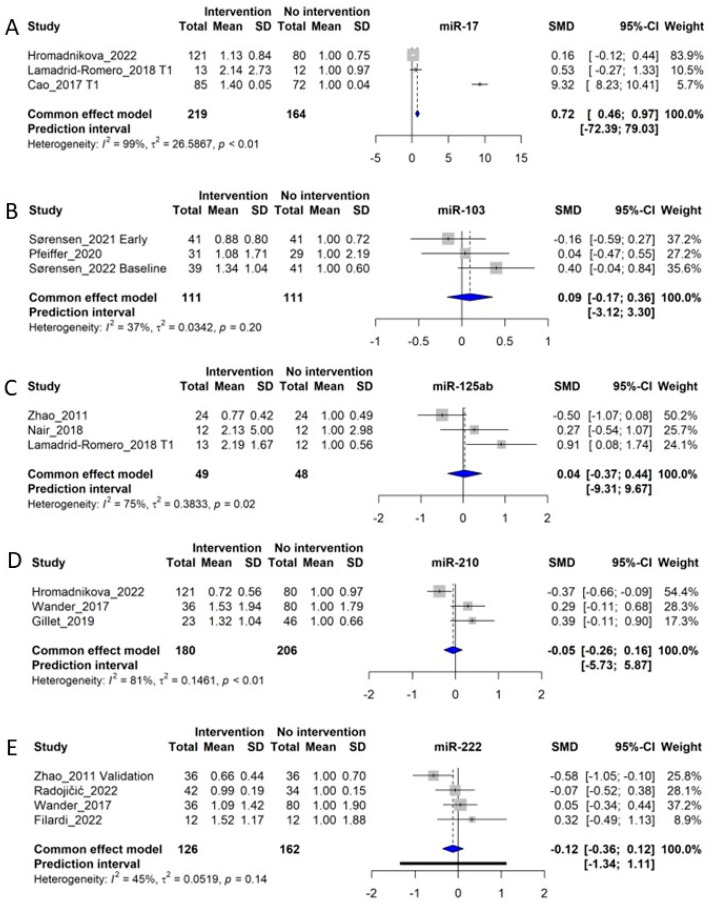
Forest plots of the fixed-effect meta-analysis of (**A**) miR-17 [30,33,37], (**B**) miR-103 [16,20,21], (**C**) miR-125a/b [18,37,52], (**D**) miR-210 [28,33,41], and (**E**) miR-222 [18,28,29,51].

miR-17 was investigated in 3 studies including 219 control subjects and 164 GDM patients [30,33,37]. The meta-analysis of the studies showed an SMD of 0.72 (Cl: 0.46–0.97, *p* < 0.05), corresponding to a fold increase of 1.72 in GDM patients, however, with a high degree of heterogeneity among studies (*I*^2^ of 99%, τ^2^ = 26.5867, and *p* < 0.01 (Figure 6A). Moreover, although the meta-analysis suggests a significant difference in the level of circulating miR-17 in a GDM pregnancy compared with a normal pregnancy, these data should be interpreted with caution, because the majority of the weight of the meta-analysis is carried by one study only [33], limiting how representative the data are.

#### 3.14.2. miR-103

We included 3 studies investigating circulating miR-103, consisting of a total of 111 control subjects and 111 GDM patients [16,20,21]. However, the meta-analysis of the studies could not demonstrate any overall difference between circulating miR-103 between NGT and GDM subjects (SMD of 0.09, CI: −3.12 to 3.30, and *p* > 0.05). The meta-analysis displays heterogeneity of *I*^2^ = 37%, τ^2^ = 0.0342, and *p* = 0.20, indicating that the insignificant overall difference between NGT and GDM is not due to outlier studies (Figure 6B).

#### 3.14.3. miR-125a and miR-125b

We included miR-125a and miR-125b in one meta-analysis due to the similarity of the miRNAs and their overall similar regulation in metabolic disease [64,65]. MiR-125a/b was investigated in 3 original studies, including 49 control subjects and 48 GDM patients, and yielded an SMD of 0.04 (CI: −0.37–0.44), indicating no differences in levels in circulation between NGT and GDM subjects [18,32,37]. The meta-analysis displays significant heterogeneity of *I*^2^ = 75%, τ^2^ = 0.3833, and *p* = 0.02 (Figure 6C). Moreover, two other studies, not included in the meta-analysis, displayed an upregulation of miR-125b in GDM [33,34]. Thus, inconsistencies between studies indicate that miR-125 species are not reliable risk indicators for GDM.

#### 3.14.4. miR-210

MiR-210 is a well-characterized ‘hypoxi-miR’, being consistently reported as upregulated by low oxygen tension [66]. As such, this miRNA could be highly relevant as an indicator of pregnancy complications. We identified three original studies investigating miR-210 for inclusion into the meta-analysis [28,33,41]. These studies included a total of 180 control subjects and 206 GDM patients. The meta-analysis indicated no overall association between levels of miR-210 and GDM diagnosis, with an SMD of −0.05 corresponding to a fold increase of 0.95 in GDM patients (CI: −0.28–0.16). The meta-analysis showed a heterogeneity of *I*^2^ = 81%, τ^2^ = 0.1461, and *p* < 0.01 (Figure 6D). Two studies, one by Gillet et al. [41] and one by Wander et al. [28], show an upregulation of miR-210, while the study of Hromadnikova et al., shows a downregulation of miR-210 [33]. Thus, we identify no overall changes in circulating levels of miR-210 in GDM patients. In patients with placental insufficiency or preeclampsia, this miRNA could be an indicator of placental oxygenation [67]; however, most included studies in this review focus on first-trimester patients, whereas placental insufficiency may not appear until later in pregnancy.

#### 3.14.5. miR-222

A total of 4 studies examined miR-222 [18,28,29,51] and included 126 control subjects and 162 GDM patients. The SMD of the meta-analysis was −0.12, corresponding to an overall fold change of 0.88 in GDM patients (CI: −0.36–0.12 *p* > 0.05). The meta-analysis had a heterogeneity of *I*^2^ = 45%, τ^2^ = 0.0519, and *p* = 0.14 (Figure 6E). Thus, circulating levels of miR-222 are not consistently associated with GDM.

### 3.15. Dependency of Gestational Time of Levels of miR-16-5p

**Figure 7:**
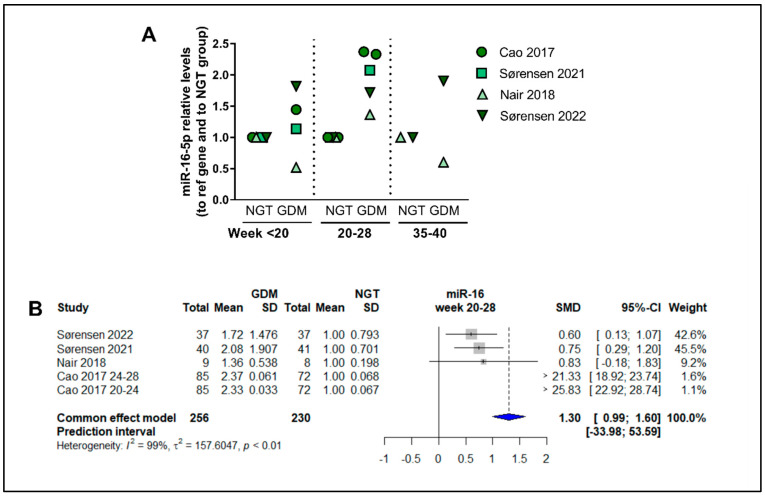
Overall temporal regulation of the circulating levels of miR-16-5p throughout gestation: (**A**) Circulating levels of miR-16-5p at <20 weeks, 20–28 weeks, and 35–40 weeks of gestation, plotted for Cao et al., 2017 [30], Sørensen et al., 2021 [21], Nair et al., 2018 [52], and Sørensen et al., 2022 [20], calculated relative to levels in nondiabetic control women matched for gestational age. (**B**) Main-effects meta-analysis for circulating levels of miR-16-5p at gestational weeks 20–28.

To analyze the possible temporal regulation of circulating miRNAs through the gestational period, we examined which miRNAs were measured at several time points in more than two studies. MiRNA-16.5p was the only miRNA fulfilling this criterion, being investigated in four studies [20,21,30,52] (Figure 7), while other miRNAs were at the most investigated for the same gestational time points in up to two studies. For miR-16-5p, circulating levels at midgestation are significantly higher (raw average of the four studies: 1.96, CI:1.44–2.51) compared with NGT subjects (Figure 7A), and the meta-analysis of the studies showed an SMD of 0.99 (Cl: 0.99–1.60, *p* < 0.05), corresponding to a fold increase of 1.99 in GDM patients, however, with a high degree of heterogeneity among studies (*I*^2^ of 99%, τ^2^ = 157.6, and *p* < 0.01 (Figure 7B). The raw average levels in early pregnancy (<20 weeks) were 1.23-fold vs. nondiabetic women, compared with the 1.96-fold at weeks 20–28, although this was not significantly different (*p* = 0.06, *n* = 4–5). Of note, one of the studies followed an apparently lower trajectory of miR-16-5p levels [52] and could be an outlier. Levels at late gestation appear to also be similar to levels in midgestation (Figure 7A).

## 4. Discussion and Conclusions

In the current systematic review and meta-analysis, we aimed to identify circulating miRNAs associated with GDM diagnosis following PRISMA guidelines. Furthermore, the aim was to perform a quantitative meta-analysis of miRNAs. Our primary literature search identified 849 articles, which we screened according to predefined inclusion and exclusion criteria to yield 35 original studies, for which we extracted quantitative data. We identified more than 100 different miRNAs investigated in relation to GDM and completed a meta-analysis for 13 different miRNAs reported in more than 3 studies. In total, 8 of these were significantly associated overall with GDM: circulating miR-29a, miR-330, miR-134, miR-16, miR-223, and miR-17 were increased in GDM, while miR-132 and miR-155 were decreased in GDM patients.

miR-29a was significantly increased in circulation in GDM (Figure 4A) but has also been reported to be associated with Type 2 diabetes in recent systematic reviews [68,69]. Hence, the upregulation of circulating miR-29a observed in multiple studies of early diagnosed GDM could also reflect pregestational impaired glucose tolerance [70]. Interestingly, acute suppression of miR-29 using an antisense inhibitor in high fat diet fed obese mice ameliorated hepatic insulin resistance and improved glucose tolerance [71], indicating a causative role in the maintenance of glucose tolerance [3].

miR-330, increased in circulation in GDM (Figure 4B), could be more specifically upregulated by GDM, as this was not reported to be associated with Type 2 diabetes [69]. miR-330 was reported to cause beta-cell dysfunction in GDM [47], as well as induce insulin resistance by causing M2 macrophage polarization [72]. Moreover, increased levels of miR-330 were also reported to be associated with decreased insulin levels [73].

miR-134, part of the imprinted MEG3-DLK genomic cluster of miRNAs on Chr. 14 controlling beta-cell function [58,74], was upregulated in circulation in GDM patients (Figure 4C). In cell culture, miR-134 levels are increased by elevated glucose concentrations (25 mM) and inhibit angiogenesis [75], as well as inducing trophoblast cell inflammation and apoptosis [17].

miR-132, increased by AMPK signaling and important for beta-cell function [76], was decreased in the circulation of GDM patients (Figure 4D) and was also reported to be associated with Type 2 diabetes [69]. miR-132 is required for beta-cell proliferation, acting via suppression of phosphatase and tensin homolog (PTEN) [77], while it is downregulated in beta-cells of obese mice [78], in concordance with the observations of circulating levels in GDM (Figure 4D).

Circulating miR-16 was increased in GDM samples (Figure 5A) and was reported, together with miR-29a and miR-134, to form a predictive biomarker algorithm for GDM [21]. Interestingly, deletion of miR-16-5p in skeletal muscle positively correlates with insulin sensitivity [79], and circulating levels of miR-16-5p are also correlated positively with insulin sensitivity [80], which is not well-reconciled with circulating miR-16-5p being increased in GDM, which is a state characterized by impaired insulin sensitivity. However, Cao et al. (2017) [30] found circulating miR-16-5p levels to be highly correlated with insulin resistance, measured with the HOMA-IR index.

miR-223 was increased in circulation in GDM (Figure 5B) but has been reported to be decreased in plasma from Type 2 diabetes patients [69]. It is therefore a possibility that miR-223 is differentially regulated in GDM compared with Type 2 diabetes. miR-223 is highly enriched in cells of hematopoietic origin, and it is conceivable that the increased miR-223 in GDM could be due to the increased body weight often observed in GDM patients, as increased body weight also increases the number of cells in circulation [81]. Moreover, insulin resistance increases miR-223 levels in white adipose tissue, where it targets GLUT4 [82].

Decreased miR-155 was associated overall with a GDM diagnosis (Figure 5C) and has been associated with inflammatory responses and endothelial dysfunction [83,84,85]. Moreover, miR-155 induction during hyperlipidemic states is necessary for compensation of pancreatic beta-cells to insulin resistance in mice, while lower levels in beta-cells are associated with diabetes [86].

miR-122 in circulation was not associated overall with a GDM diagnosis (Figure 5D), although this was reported to be associated with Type 2 diabetes [63,69] with hepatic steatosis [62] and the metabolic syndrome [87].

Circulating miRNAs miR-17, -103, -125 a and -125b, -210, and -222 were also reported to have been previously investigated in relation to GDM, although none of these were consistently changed in GDM patients (Figure 6).

There are several limitations to the performed systematic review and meta-analysis: Biomarker studies are often affected by publication bias, in that insignificant associations are less reported than significantly different associations. For the systematic review, we included only published studies, hence potentially excluding studies demonstrating insignificant differences between GDM and NGT. To minimize publication bias, we included all data, including Supplementary Materials, from the included studies, regardless of whether the miRNAs displayed significant differences between groups. For the meta-analysis, it was possible to include only circulating miRNAs, for which at least three studies reported data with averages and estimates of variability, necessary for the computation of the standardized mean difference (SMD). We observed 92 circulating miRNAs, which had only each entered 1 published study. Thus, it is likely that more circulating miRNAs can be identified that associate with GDM. It is therefore possible that at least some of the miRNAs are under-represented. However, we minimized publication biases by also including data from all Supplementary Materials available. Moreover, for most of the investigated miRNAs in the meta-analysis, we identified significant heterogeneity among reported associations of the miRNAs investigated in the individual studies regarding GDM. There are several possible explanations for the observed heterogeneity: study populations may vary by ethnicity, age, time of sampling during gestation, and the sample material investigated. All these factors are likely to influence measured levels of miRNA [56].

Moreover, we also identified diversity in the measurement and normalization strategies for quantification of miRNAs: There was marked heterogeneity, among which miRNAs were investigated with respect to GDM. Of the 131 investigated miRNAs, the majority, 92 miRNAs, had only been investigated in 1 study. The 13 miRNAs that were analyzed in the ensuing meta-analysis constitute only a small fraction of the possible circulating miRNAs that could potentially be biomarkers for GDM. While we analyzed 13 miRNAs in separate meta-analyses, only 7 of these were significantly associated overall with GDM. These observations indicate that a selection bias exists, of which miRNAs are investigated in relation to GDM, which is emphasized by the observation that only a few of the included studies were based on hypothesis-free approaches, such as arrays, PCR-arrays or small RNA-sequencing for selection. Different selections of PCR normalization strategy constitutes another source of heterogeneity among studies. Although a few reference genes are more used than others (such as U6 and artificial RNA spike-ins), a large number of different targets are chosen as references. Thus, as also discussed in consensus statements for the clinical use of qPCR assays [11,56], different choices of reference genes will introduce variability between studies. Thus, standardization of assays and reporting for circulating miRNA levels would decrease between-study variability and facilitate interstudy comparisons.

While the research field of circulating miRNAs as biomarkers is not well-developed, our meta-analysis clearly shows that circulating levels of several miRNAs are significantly associated with a GDM diagnosis, despite the number of studies being low and the total number of included subjects also being low: between 50 and 350 in each group. Thus, there is a clear need for additional and larger studies of circulating miRNAs in GDM to determine with better precision the association and risk-prediction benefits that can be obtained by the inclusion of circulating miRNAs for early detection of GDM. Moreover, based on comparison with miRNAs recently reported in a systematic review for miRNAs in relation to Type 2 diabetes, it is also evident that some of the miRNAs associated with GDM (miR-29a, -132, -155, -210, and -223) are also associated with Type 2 diabetes, although the direction of change is not always the same [69]. Thus, some of the identified miRNAs may not be specific for GDM but may also be changed in pregestational hyperglycemia. Thus, to enable translation of miRNAs into clinical practice, we specifically recommend that the circulating miRNAs identified in the meta-analysis (miR-29a, miR-330, miR-134, miR-132, miR-16, miR-223, and miR-155) be tested in several large cohorts of well-characterized GDM patients and glucose-tolerant pregnant women using the same well-defined clinically validated assays using prespecified data analytical pipelines [56]. This will further allow the robust combination of several miRNAs into one biomarker profile with clear association and higher specificity for GDM, as described for miR-29a, -16, and -134 [21]. To further enable the use of circulating miRNAs as biomarkers in general, it would also be necessary to establish population baseline levels for the relevant miRNAs, as these currently remain uncharacterized.

## Figures and Tables

**Table 1:** Studies included in the meta-analysis part of the systematic review.

Study	Title	Reference	Meta-Analysis
Abdeltawab, A. et al. (2021)	Circulating micro RNA-223 and angiopoietin-like protein 8 as biomarkers of gestational diabetes mellitus	[23]	√
Cao, Y.L. et al. (2017)	Plasma microRNA-16-5p, -17-5p and -20a-5p: Novel diagnostic biomarkers for gestational diabetes mellitus	[30]	√
Filardi, T. et al. (2022)	Identification and Validation of miR-222-3p and miR-409-3p as Plasma Biomarkers in Gestational Diabetes Mellitus Sharing Validated Target Genes Involved in Metabolic Homeostasis	[51]	√
Gillet, V. et al. (2019)	miRNA Profiles in Extracellular Vesicles from Serum Early in Pregnancies Complicated by Gestational Diabetes Mellitus	[41]	√
Hocaoglu, M. et al. (2019)	Differential expression of candidate circulating microRNAs in maternal blood leukocytes of the patients with preeclampsia and gestational diabetes mellitus.	[43]	√
Hromadnikova, I. et al. (2022)	Cardiovascular Disease-Associated MicroRNAs as Novel Biomarkers of First-Trimester Screening for Gestational Diabetes Mellitus in the Absence of Other Pregnancy-Related Complications	[33]	√
Juchnicka, I. et al. (2022)	miRNAs as Predictive Factors in Early Diagnosis of Gestational Diabetes Mellitus.	[22]	√
Ke, W. et al. (2022)	miR-134-5p promotes inflammation and apoptosis of trophoblast cells via regulating FOXP2 transcription in gestational diabetes mellitus	[17]	√
Lamadrid-Romero, M. et al. (2018)	Central nervous system development-related microRNAs levels increase in the serum of gestational diabetic women during the first trimester of pregnancy.	[37]	√
Martínez-Ibarra, A. et al. (2019)	Unhealthy Levels of Phthalates and Bisphenol A in Mexican Pregnant Women with Gestational Diabetes and Its Association to Altered Expression of miRNAs Involved with Metabolic Disease	[9]	√
Nair, S. et al. (2018)	Human placental exosomes in gestational diabetes mellitus carry a specific set of miRNAs associated with skeletal muscle insulin sensitivity	[52]	√
Nair, S. et al. (2021)	Extracellular vesicle-associated miRNAs are an adaptive response to gestational diabetes mellitus	[36]	√
Pfeiffer, S. et al. (2020)	Circulating miR-330-3p in Late Pregnancy is Associated with Pregnancy Outcomes Among Lean Women with GDM	[16]	√
Radojičić, O. et al. (2022)	Gestational Diabetes is Associated with an Increased Expression of miR-27a in Peripheral Blood Mononuclear Cells	[29]	√
Sørensen, A.E. et al. (2021)	The Predictive Value of miR-16, -29a and -134 for Early Identification of Gestational Diabetes: A Nested Analysis of the DALI Cohort	[20]	√
Sørensen, A.E. et al. (2022)	The Temporal Profile of Circulating miRNAs during Gestation in Overweight and Obese Women with or without Gestational Diabetes Mellitus	[20]	√
Wander, P.L. et al. (2017)	Circulating early- and mid-pregnancy microRNAs and risk of gestational diabetes	[28]	√
Xiao, Y. et al. (2020)	MiR-330-3p contributes to INS-1 cell dysfunction by targeting glucokinase in gestational diabetes mellitus.	[47]	√
Ye, Z. et al. (2022)	Plasma Exosomal miRNAs Associated with Metabolism as Early Predictor of Gestational Diabetes Mellitus	[19]	√
Yoffe, L. et al. (2019)	Early diagnosis of gestational diabetes mellitus using circulating microRNAs	[31]	√
Zhao, C. et al. (2011)	Early second-trimester serum miRNA profiling predicts gestational diabetes mellitus	[18]	√
Zhou, X. et al. (2019)	miR-132 serves as a diagnostic biomarker in gestational diabetes mellitus and its regulatory effect on trophoblast cell viability	[38]	√

**Table 2:** Studies included only in the qualitative systematic review.

Study	Title	Reference
Bhushan, R. et al. (2022)	MicroRNA-7 Regulates Insulin Signaling Pathway by Targeting IRS1, IRS2, and RAF1 Genes in Gestational Diabetes Mellitus	[46]
Li, Y. et al. (2021)	Study of serum miR-518 and its correlation with inflammatory factors in patients with gestational diabetes mellitus complicated with hypertensive disorder complicating pregnancy	[42]
Niu, S. et al. (2022)	The Expression and Clinical Value of miR-221 and miR-320 in the Plasma of Women with Gestational Diabetes Mellitus	[35]
Peng, H.Y. et al. (2018)	High glucose induces dysfunction of human umbilical vein endothelial cells by upregulating miR-137 in gestational diabetes mellitus	[25]
Shen, H. et al. (2021)	miR-181d promotes pancreatic beta cell dysfunction by targeting IRS2 in gestational diabetes mellitus	[45]
Stirm, L. et al. (2018)	Maternal whole blood cell miRNA-340 is elevated in gestational diabetes and inversely regulated by glucose and insulin	[24]
Tagoma, A. et al. (2018)	MicroRNA profiling of second trimester maternal plasma shows upregulation of miR-195-5p in patients with gestational diabetes.	[34]
Wang, F. et al. (2021)	Circulating miRNAs miR-574-5p and miR-3135b are potential metabolic regulators for serum lipids and blood glucose in gestational diabetes mellitus	[40]
Wei, L. et al. (2021)	Elevated Serum and Urine MiR-429 Contributes to the Progression of Gestational Diabetes Mellitus	[27]
Wen, J. et al. (2021)	miR-520h Inhibits cell survival by targeting mTOR in gestational diabetes mellitus	[44]
Yu, X. et al. (2021)	miR-96-5p: A potential diagnostic marker for gestational diabetes mellitus	[39]
Zhang, L. et al. (2021)	Diagnostic value of dysregulated microribonucleic acids in the placenta and circulating exosomes in gestational diabetes mellitus	[53]
Zhang, Y.L. et al. (2020)	Dysregulation of microRNA-770-5p influences pancreatic-β-cell function by targeting TP53 regulated inhibitor of apoptosis 1 in gestational diabetes mellitus	[48]

## Data Availability

All data are included in Supplementary Table S1.

## Supplementary Materials

The following supporting information can be downloaded at: https://www.mdpi.com/article/10.3390/ijms24076186/s1.
